# On Chloroform

**Published:** 1848-03

**Authors:** J. Y. Simpson

**Affiliations:** Philadelphia


### MEDICAL EXAMINER,

AND

### RECORD OF MEDICAL SCIENCE.

NEW SERIES.—No. XXXIX.—MARCH, 1848.

---

### ORIGINAL COMMUNICATIONS.

---

### *On Chloroform.* By Professors Simpson and Meigs.

We have been favoured by Professor Meigs with the following letter from Dr. Simpson, and his reply. A correspondence between these two eminent teachers of obstetrics, on the use of this new agent in the practice of midwifery, will be read with deep interest.

Edinburgh, January 23rd, 1848.

*Dear Sir,*—By private letters from America, brought by the last steamer, I hear that in most of the cities of the Union, your chemists had failed in preparing proper chloroform ; and that consequently, most experiments tried with it, had been unsuccessful. In Great Britain and on the Continent of Europe, chloroform has every where entirely, or nearly entirely, superseded the use of sulphuric ether, as an anæsthetic agent. The want of success which has attended its employment in America, is, perhaps, owing in a great measure to an error of my own, viz. : to my not stating in my original account of it, the proper method of purifying it. This and other omissions were owing to the haste with which my first paper was drawn up.

I will feel, therefore, deeply obliged by your taking any measures that you may deem fit, to circulate amongst American medical men, the formula which I inclose for the preparation of chloroform. It is the formula used by Messrs. Duncan and Flockhart, our Edinburgh druggists, who have already manufactured enormous quantities of it. They always now are able to produce it as heavy as 1500 in specific gravity. Their first distillation of it is made in two large wooden barrels, with a third similar barrel as a receiver. They throw hot steam into the two first barrels, which serves to afford both sufficient heat and water for the process. They employ sixty pounds of chloride of lime at each distillation, and have been able to manufacture three hundred ounces of chloroform a day. Each ounce of the chloride yields, in the long run, about half an ounce of chloroform: consequently, to obtain three hundred ounces, (as above) about six hundred ounces of bleaching powder are required. At first they could only make ten or twenty ounces per diem, then they rose to sixty, and latterly, enlarging their barrels, they can make, as I have said, three hundred ounces in the twenty-four hours.

Various other chemical houses in Edinburgh, Liverpool, Glasgow, York, London, &c., are busy manufacturing it, in great quantities. They keep their formulas as secrets. But none of them make so good an article as Duncan & Flockhart, whose formula I append.

The statements which I have already made, may show you to what an extent the chloroform is used in this country; and our chemists tell me that the demand for it steadily increases with them.

In *Surgery*, its use is quite general, for operations, painful diagnosis, &c. My friend, Dr. Andrew Wood, has just been telling me of a beautiful application of it. A boy fell from a height, and severely injured his thigh. It was so painful that he shrieked when Dr. Wood tried to handle the limb; and would not allow of a proper examination. Dr. W. immediately chloroformed him—at once ascertained that the femur was fractured—kept him anæsthetic till he sent for his splints—and did not allow his patient to awake till his limb was all properly set, bandaged, and adjusted.

In *Medicine* its effects are being extensively tried as an anodyne, an anæsthetic, a diffusible stimulant, &c. Its anti-spasmodic powers in colic, asthma, &c., are every where recognized.

In *Midwifery*, most or all of my brethren in Edinburgh employ it constantly. The ladies themselves, insist on not being doomed to suffer, when suffering is so totally unnecessary. In London, Dublin, &c., it every day gains converts to its obstetric employment, and I have no doubt that those who most bitterly oppose it now, will be yet, in ten or twenty years hence, amazed at their own professional cruelty. They allow their medical prejudices to smother and over-rule the common dictates of their profession, and of humanity.

No accidents have as yet happened under its use, though several hundred thousand must have already been under the influence of chloroform. Its use here has been a common amusement in drawing-room parties, for the last two or three months.

I never now apply it with anything but a silk handkerchief. In surgical cases and operations, the quantity given is not in general measured. We all judge more by the *effects* than the *quantity*. Generally, I believe, we pour two or three drachms on the handkerchief at once, and more in *a minute*, if no sufficient effect is produced, and we stop when sonorous respiration begins. Not unfrequently spasms, rigidity, &c., come on, but they disappear as the effect increases, and none of us care for them any more than for hysteric symptoms; nor do they leave any bad effect. But the mere *appearance* of them is enough to terrify a beginner.

I shall be glad to hear how the cause of anæsthesia gets on among you, and I remain with great respect,

Very faithfully yours,

J. Y. Simpson.

To Professor Meigs.

---

The following is the formula for Chloroform, communicated by Professor Simpson:

| | | |
|---|---|---|
| Take of Chloride of Lime in powder, | | 4 pounds. |
| Water, | . . . . . | 12 " |
| Rectified Spirit, | . . | 12 fluid ounces. |
| | | "Dumas." |

The chloride of lime and water being first well mixed together, the spirit is added. Heat is then applied to the still, (which ought not to be more than a third full,) but as soon as the upper part of the still becomes warm, the heat is withdrawn and the action allowed to go on of itself. In a short time the distillation commences, and whenever it begins to go on slowly the heat is again applied. The fluid which passes over separates into two layers, the lower of which is Chloroform. This, after having been separated from the weak spirit forming the upper layer, is purified by being mixed with half its measure of strong sulphuric acid, added gradually. The mixture, when cool, is poured into a leaden retort, and distilled from as much carbonate of baryta by weight, as there is of sulphuric acid by measure. The product should be allowed to stand over quicklime for a day or two, and repeatedly shaken and then redistilled from the lime.

---

*Dr. Meigs' Reply to Professor Simpson's Letter.*

PHILADELPHIA, Feb. 18th, 1848.

*Dear Sir*,—I have to acknowledge the favour of your letter of Jan. 23d, which I received yesterday.

The chemists in this country have produced very perfect chloroform, of the specific gravity of 1450, as I am informed, and which is much employed in dentistry operations, and to a considerable extent also in surgery.

I presume you will, ere this date, have received copies of Prof. Warren's pamphlet on "Etherization," which may inform you, very fully, as to the use of the anæsthetic agent in the Massachusetts General Hospital and in Boston. That eminent gentleman is more reserved as to the obstetric employment of the agent; much more so, I understand, than either Dr. Channing, Dr. Homans, and other practitioners, who make use of it very commonly.

In New York, as I learn, the surgical application of chloroform is common, while its obstetrical use has not as yet acquired a general vogue.

In Philadelphia, we have the Pennsylvania Hospital, with more than two hundred beds. A very considerable amount of surgical practice, which renders that house a favourite clinical study for medical students of the United States, has not, as yet, furnished a single example of the exhibition of chloroform or ether as anæsthetic agents. The Surgical staff of the Institution have not become convinced of the propriety of such a recourse in the operations performed there.

In the Jefferson College, to which I am attached, as Professor of Midwifery, etc., there is a medical and surgical Clinic held on the Wednesday and Saturday in each week. The resort of surgical cases there is very great, and a Clinical day rarely passes without some some surgical operations before the Classes. The clinical professors, (in surgery) Drs. Mütter and Pancoast, almost invariably employ the chloroform, and the successful exhibition of the article has entirely confirmed them in their opinion of its great value. Some of the operations have been of the gravest character, and no serious event has occurred to check the career of the remedy.

As to its employment in Midwifery here, notwithstanding a few cases have been mentioned and reported, I think it has not yet begun to find favour with accoucheurs.

I have not exhibited it in any case; nor do I, at present, know of any intention in that way, entertained by the leading practitioners of obstetrical medicine and surgery, in this city. I have not yielded to several solicitations as to its exhibition addressed to me by my patients in labour.

As to the extension of the anæsthesia in the Southern and Western States, I am not at present enabled to give you information. I believe the practice is slowly gaining converts, and that it will become more and more common ere long.

You may perhaps feel surprised at this admission on my part, seeing that I am still a recusant; and I ought therefore to be allowed to explain myself, lest I should continue to appear unreasonable in your eyes.

Having carefully read the *Comptes Rendus* of the Royal Academy of Medicine of Paris, which contained full Reports of the copious discussions on the question of the Letheon, a few months since, and having also seen the English and American Reports in the Journals, and particularly having read your own pamphlet of "Remarks," &c., I may not properly be accused of ignorance of the power, effects, or motives, in relation to chloroformization in surgery or obstetricy. The copy of your own pamphlet, for which I now beg leave to thank you, would necessarily have put me *au niveau* on the subject.

Not being myself engaged in the practice of surgery, proper, I prefer to avoid any expression of opinion as to the propriety of the practice; and I do this upon the principle, *suum cuique tributo.* It would be an impertinence in me, were I to interfere with the conduct of the surgeons.

But, in midwifery, to which a long and extensive practice has enured me, and rendered me a familiar, dispassionate witness of its various forms and phenomena, I am less liable to misconceptions. And here allow me to say, I have been accustomed to look upon the sensation of pain in labour as a physiological relative of the power or force; and notwithstanding I have seen so many women in the throes of labour, I have always regarded a labour-pain as a most desirable, salutary, and conservative manifestation of life-force. I have found that women, provided they were sustained by cheering counsel and promises, and carefully freed from the distressing element of terror, could in general be made to endure without great complaint, those labour-pains which the friends of the anæsthesia desire so earnestly to abolish and nullify for all the fair daughters of Eve.

Perhaps, dear sir, I *am* cruel in taking so dispassionate a view of the case; and it is even possible that I may make one of the number of those "amazed" converts of whom you speak in your worthy letter to me. But, for the present, regarding the pain of a Natural labour as a state not, by *all* possible means and always, to be eschewed and obviated, I cannot bring myself to the conviction that of the two, whether labour-pain or insensibility, insensibility is to be preferred.

If I could believe that *chloroformal* insensibility is sleep indeed, the most considerable of my objections would vanish. Chloroform is not a soporific; and I see in the anæsthesia it superinduces a state of the nervous system in no wise differing from the anæsthetic results of alcoholic potations, save in the suddenness and transitiveness of its influence.

I freely admit, for I know it, that many thousands of persons are daily subjected to its power. Yet I feel that no law of succession of its action on the several distinct parts of the brain has been or can be hereafter ascertained, seeing that the succession is contingent. Many grave objections would perhaps vanish could the law of the succession of influences on the parts of the brain be clearly made out and its provisions ensured There are, indubitably, certain cases in which the intellectual hemispheres are totally hebetized and deprived of power by it, while the co-ordinating lobes remain perfectly unaffected. In others the motor cords of the cerebro-spinal nerves are deprived of power, whilst the sensitive cords enjoy a full activity, and vice versâ.

In some instances, the seeing brain enables the patient to look upon the application of a cautery that he does not feel while it sears him, or of a bistoury, whose edge gives him no pain. In others, the influence of the agent upon the sources of the pneumogastric and phrenic nerves is dangerously, or at least alarmingly, made manifest by modifications of the respiratory force. It appears to me therefore quite certain that there is no known law of succession of the ether-influences on the several parts of the brain. It is known that the continued aspiration of the vapour brings at last the medulla oblongata fully under its anæsthetic power, and the consequent cessation of respiration, which determines the cessation of the oxygenation of the blood, and thereby of the brain, is death. M. Flourens' experiments, and others, especially those by the younger Mr. Wakley, of the Lancet, prove very conclusively that the aspiration of ether or chloroform, continued but a little longer than the period required for hebetizing the hemispheres, the cerebellum, the tubercula quadrigemina, and the cord, overthrows the medulla oblongata, and produces thereby sudden death. I fully believe with M. Flourens that the medulla oblongata is the *nœud vital*, and that, though later brought under the power of chloroformization, it is always reducible under it. Hence I fear that in all cases of chloroformal anæsthesia, there remains but one irrevocable step more to the grave.

I readily hear, before your voice can reach me across the Atlantic, the triumphant reply that an hundred thousand have taken it *without accident!* I am a witness that it is attended with alarming accidents, however rarely. But should I exhibit the remedy for pain to a thousand patients in labour, merely to prevent the physiological pain, and for no other motive—and if I should in consequence destroy only one of them, I should feel disposed to clothe me in sack-cloth, and cast ashes on my head for the remainder of my days. What sufficient motive have I to risk the life or the death of one in a thousand, in a questionable attempt to abrogate one of the general conditions of man?

As to the uses of chloroform in the medical or therapeutical treatment of pain, the question changes. There is no reasonable therapia of health. Hygieinical processes are good and valid. The sick need a physician, not they that are well. To be in natural labour is the culminating point of the female somatic forces. There is, in natural labour, no element of disease—and, therefore, the good old writers have said nothing truer nor wiser than their old saying, that "*a meddlesome midwifery is bad.*" Is chloroformization meddlesome?

Your countryman, old Thomas Rainold, in "the Woman's Booke, or The byrthe of Mankynde, at fol. LIII. says, "Very many be the perilles, daungers, and thronges, which chaunce to women in theyr labour." These are the cases requiring our therapeutical and chirurgical intervention. You will, my dear sir, think me a hopeless recusant, if I decline the anæsthesia here also. I pray you, therefore, allow me to state my reasons for such recusancy.

If I were amputating a limb, or extirpating a tumour, I should see all the steps of my incisions, ligations, &c. But if I apply my forceps in a right occipito-posterior position, (fourth of Baudeloque,) I know that I thrust the blade of the male branch, far upwards betwixt the face of the child and the upper third of the vagina, which, in this case, is already greatly expanded, and that the extremity of the blade is separated from the peritoneum, only by the mucous and condensed cellular coat of the tube. Now, no man, can *absolutely* know the precise degree of inclination his patient will give to the plane of her superior strait, while in pain; an inclination to be modified by every movement of her body and limbs. Under such absolute uncertainty, the best guide of the accoucheur is the reply of the patient to his interrogatory, "Does it hurt you?" The patient's reply, "Yes and no," are worth a thousand dogmas and precepts, as to planes and axes, and curves of Carus. I cannot, therefore, deem myself justified in casting away my safest and most trustworthy diagnosis, for the questionable equivalent of ten minutes exemption from a pain, which, even in this case, is a physiological pain.

Having thus, in my own defence, and not as attacking your opinion, set forth the motives that have hitherto served to restrain me from the administration of chloroform, I desist from giving you any further trouble in this line of thought. I have, sir, a far more pleasing duty to perform, in saying that your name is as well known, perhaps, in America as in your native land, and to congratulate you on the extension of your fame. I had the pleasure to read your interesting letter to my class, consisting of several hundred young gentlemen, who listened to your words with the same respect, as they would have paid to you, had they been pronounced by your own lips. They will disperse themselves in a few days hence, over all the States of the Union, and thus will have it in their power to report the latest dates of your opinions as to chloroform. I shall also allow it to be published on the first proximo, in a medical journal of extensive circulation. You will herein perceive the readiness with which I assist in disseminating your views. It is not without regret that I find myself opposed to your opinions in the case. That difference ought not, however, in the least degree to affect those sentiments of respectful consideration and real esteem with which I am, dear sir, very faithfully, your obedient servant,

Ch. D. Meigs.

Professor Simpson, &c.

